# The Stability Prediction and Epitaxial Growth of Boron Nitride Nanodots on Different Substrates

**DOI:** 10.3390/molecules29061313

**Published:** 2024-03-15

**Authors:** Muhamad Jalu Purnomo, Yosi Febrita, Okto Dinaryanto, Wojciech Gierlotka, Ing-Song Yu

**Affiliations:** 1 Department of Materials Science and Engineering, National Dong Hwa University, Hualien 97401, Taiwan; jalu_p@yahoo.com (M.J.P.); yosi_febrita@yahoo.com (Y.F.); 2 Institut Teknologi Dirgantara Adisutjipto, Yogyakarta 55198, Indonesia; okto.dinaryanto@mail.ugm.ac.id

**Keywords:** boron nitride, cohesive energy, density functional theory, nanodot, molecular beam epitaxy

## Abstract

Boron nitride (BN) is a wide-bandgap material for various applications in modern nanotechnologies. In the technology of material science, computational calculations are prerequisites for experimental works, enabling precise property prediction and guidance. First-principles methods such as density functional theory (DFT) are capable of capturing the accurate physical properties of materials. However, they are limited to very small nanoparticle sizes (<2 nm in diameter) due to their computational costs. In this study, we present, for the first time, an important computational approach to DFT calculations for BN materials deposited on different substrates. In particular, we predict the total energy and cohesive energy of a variety of face-centered cubic (FCC) and hexagonal close-packed (HCP) boron nitrides on different substrates (Ni, MoS_2_, and Al_2_O_3_). Hexagonal boron nitride (h-BN) is the most stable phase according to our DFT calculation of cohesive energy. Moreover, an experimental validation equipped with a molecular beam epitaxy system for the epitaxial growth of h-BN nanodots on Ni and MoS_2_ substrates is proposed to confirm the results of the DFT calculations in this report.

## 1. Introduction

Boron nitride (BN) is a chemically stable material within group III-V compounds, particularly applied in electronic and optoelectronic devices [1,2,3]. The crystal structure polymorphism of BN includes hexagonal (h-BN), rhombohedral (r-BN), turbostratic (t-BN), wurtzite (w-BN), and cubic (c-BN) structures. h-BN shares a similar structure to graphene with a 1.7% lattice mismatch, making it a two-dimensional (2D) material. The outstanding physical properties of h-BN include high thermal conductivity and a wide bandgap. h-BN serves not only as a 2D material for electronic devices as an insulating layer or quantum tunnel barrier but also holds high potential for deep-ultraviolet light-emitting diode (LED) applications. The second popular phase of BN is a cubic structure, like a diamond, which emerges under high-temperature and high-pressure conditions from h-BN. Cubic BN boasts wide indirect bandgaps with energy levels of 6.4 eV [4]. It is now highly sought after for applications such as coatings on cutting tools, wear- and corrosion-resistant coatings, heat sinks, and high-temperature electronic devices [5]. Nanodots (NDs) are standalone nanoparticles with nanometer-scale dimensions. Due to the quantum confinement effect, NDs exhibit distinct optical and electronic properties compared with larger particles. BN nanostructures are of great interest and have applications in many fields based on their amazing properties [6].

Various techniques have been employed for the fabrication of BN, including physical vapor deposition (PVD) [7,8,9,10], chemical vapor deposition (CVD) [11,12,13,14], and molecular beam epitaxy (MBE) [15,16,17,18], to grow both c-BN and h-BN. Among these methods, plasma-assisted molecular beam epitaxy (PA-MBE) allows for precise growth control, resulting in high-quality epitaxy at lower growth temperatures [19]. BN has also garnered attention for its growth on various substrates, such as Ni [20], cobalt [21], and sapphire (Al_2_O_3_) [22]. Stable growth conditions are crucial for the successful deposition of boron nitride (BN) on substrates [23]. When BN growth is stable, it typically indicates a good match in surface energies with the substrate, ensuring uniform nucleation and reduced defect formation. While stability prediction is one of the main issues in the BN fabrication techniques, research in this area is still relatively scarce. Therefore, the stability prediction of BN needs to be investigated experimentally and computationally.

In materials science, computational calculations are indispensable prerequisites for experimental works, enabling precise property prediction and experimental guidance. Quantum-mechanical simulations based on density functional theory (DFT) represent an effective means for prediction and characterization at the atomistic scale for several properties of materials [24,25]. The broader community increasingly acknowledges the detailed chemical and physical insights provided by first-principles theoretical simulations, so the computational approach has recently become a standard complementary tool to experimental characterization in many research fields.

Density functional theory holds a central position in predicting essential properties like stability crystals, crystal structures, lattice constants, and bond lengths [26]. DFT calculations are often used to validate experimental results [27], as demonstrated by A. A. Tonkikh et al.’s agreement between DFT calculations and experimental data for the bulk electronic properties of h-BN [17]. Restuccia et al. used ab initio methods to determine the adhesion energy of metallic heterostructures [28]. DFT also can aid in identifying stable crystal configurations and exploring novel materials [29]. Several widely used software packages facilitate DFT calculations, including VASP version 5.2.12 [30,31], Quantum ESPRESSO v. 6.4 [32,33], ABINIT at latest version of December 2000 [34], Gaussian 03 [35,36], CASTEP cerius^2^ version 2.0 [37], and WIEN2k version 19 [38]. VASP is widely used because of its accuracy, efficiency, and versatility, making it a leading choice for predicting crystal stability through DFT calculations in materials science [39]. Researchers rely on DFT to calculate physical and chemical properties, including cohesive energy, which helps predict the stability of the material. Cohesive energy is the amount of energy required to disassemble a solid material into its constituent atoms or molecules. It quantifies the strength of the bonds that hold the material together. The energy between the deposited material and the substrate, commonly known as “cohesive energy” or “adhesion energy”, is vital because it influences the quality of thin films or nanostructures, affecting its adhesion to the substrate, structural integrity, and performance in its final application. Lower (more negative) cohesive energy indicates greater stability, while higher cohesive energy suggests reduced stability and a propensity for disintegration [40].

In the field of boron nitride (BN) research, while significant progress has been made in understanding its diverse applications and crystal structures, there remains a notable research gap regarding the stability prediction of BN on various substrates. Stable growth conditions are essential for successful BN deposition, yet relatively little research has been conducted to predict and understand stability in this context. This gap presents a critical opportunity for both experimental and computational investigation.

In this study, we aim to address this research gap by employing a comprehensive approach that integrates computational predictions using density functional theory (DFT) calculations with experimental validation through plasma-assisted molecular beam epitaxy (PA-MBE) growth. Our primary focus is on predicting the stability of BN on different substrates, including Ni, MoS_2_, and Al_2_O_3_, through precise DFT calculations. Additionally, we propose an experimental growth method by using PA-MBE on polycrystalline nickel and few-layer 2D MoS_2_/sapphire substrates to validate our computational predictions. Furthermore, we aim to bridge the gap between computational predictions and experimental observations by conducting detailed characterizations of the experimental samples using field-emission scanning electron microscopy (FE-SEM), atomic force microscopy (AFM), Raman spectroscopy, and transmission electron microscopy (TEM). By investigating the influence of atomic structure optimization and the selective dynamics of BN and substrate atoms, we seek to provide valuable insights into the factors influencing BN stability on different substrates. This comprehensive approach, combining computational modeling and experimental validation, will contribute to advancing our understanding of BN stability and pave the way for the informed design and optimization of BN-based materials for various applications in electronics, optoelectronics, and beyond.

## 2. Results and Discussion

### 2.1. DFT Approach

**Figure 1:**
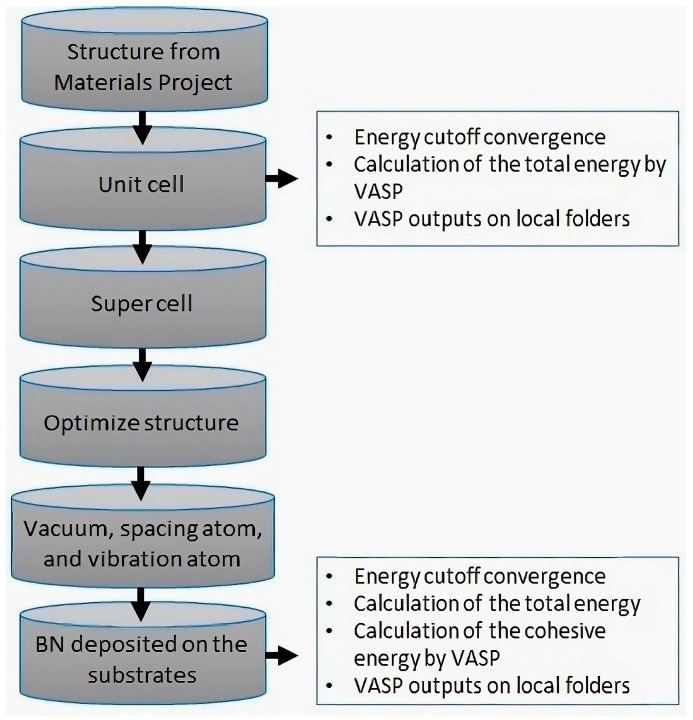
Flow diagram explaining the sequence of logical steps followed by our computational workflow to calculate the properties of the solid interface.

Density functional theory (DFT) serves as an indispensable predictive tool for guiding experimental endeavors, offering unparalleled computational efficiency and accuracy in simulating electronic structures and properties. By deploying DFT analyses before experimental trials, researchers can significantly mitigate risks, optimize resource allocation, and forecast outcomes with a precision often unattainable through empirical methods alone. Consequently, DFT has evolved into a cornerstone of computational physics and chemistry, fostering significant advancements across diverse scientific disciplines by bridging theoretical and experimental domains. Before initiating DFT calculations, the preliminary step involves preparing the atomic structures of BN and various substrates, including Ni, MoS_2_, and Al_2_O_3_. Once these atomic structures are prepared, the next course of action is to carry out atomic optimization for each of these structures. This optimization aims to determine the most stable atomic configurations. Subsequently, the formation of a supercell for the atomic structures is undertaken before entering the computational phase. These computational steps are executed using the Vienna Ab Initio Simulation Package (VASP) software to calculate the structure’s total energy, per the procedure illustrated in Figure 1.

#### 2.1.1. Optimization of the Structures

In this study, three different substrates—Ni, MoS_2_, and Al_2_O_3_—as well as BN in two crystal structures, were utilized. Therefore, prior to computing cohesive energy, all structures underwent relaxation. Since the supercells were constructed from unit cells, relaxing a single unit cell from each phase mentioned above was sufficient. Subsequently, supercells comprising substrates and depositions were created, and the energies of these structures were computed. The sizes of the supercells employed in this study are detailed in Table 1. Additionally, the lattice parameters of the substrates were determined to be the following lengths: *a* ≈ 8.602 Å, *b* ≈ 7.440 Å, and *c* ≈ 30.000 Å.

Each substrate (Ni, MoS_2_, or Al_2_O_3_) was paired with both structures of BN (hexagonal and cubic) in a manner such that the deposition was positioned above the 111 crystal plane due to the similar size of the crystalline lattices of the deposition and substrate. Following this, the structure composed of the substrate and deposition underwent further relaxation—allowing the two top layers and deposition to move freely—which facilitated the attainment of a stable crystal structure. The energy calculated at this stage was subsequently utilized to determine the cohesion energy according to Equation (4) (in Section 3.1).

**Figure 2:**
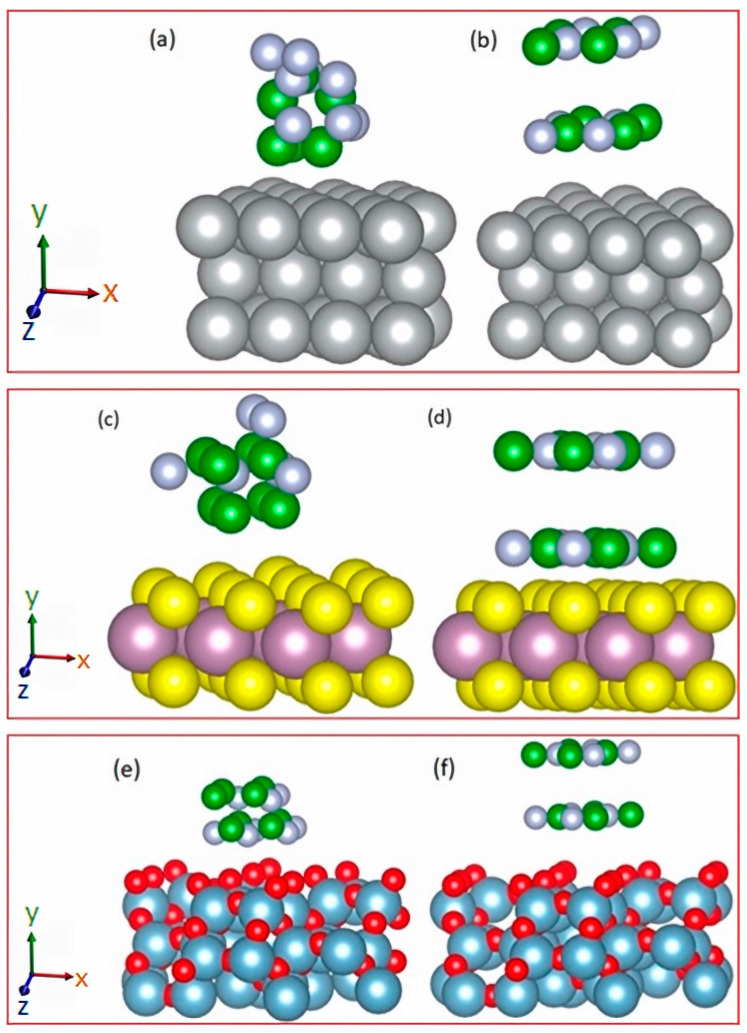
Illustration for the crystal structures of BN nanodots grown on different substrates: (**a**) c-BN grown on Ni, (**b**) h-BN grown on Ni, (**c**) c-BN grown on MoS_2_, (**d**) h-BN grown on MoS_2_, (**e**) c-BN grown on Al_2_O_3_, and (**f**) h-BN grown on Al_2_O_3_.

The six possible configurations of BN/substrates are illustrated in Figure 2a–f.

#### 2.1.2. Total Energy

The results of DFT calculations for each element and structural configuration are presented in Table 2. Before configuring BN’s growth on various substrates, the initial step involves calculating the total energy of each component, including cubic BN (c-BN), hexagonal BN (h-BN), nickel (Ni), molybdenum disulfide (MoS_2_), and aluminum oxide (Al_2_O_3_) structures. Subsequently, the total energy is computed for structural configurations of c-BN grown on several substrates, followed by the same procedure for h-BN configurations on multiple substrates. Once the total energy values for each structural configuration are obtained, Equation (4) determines the cohesive energy.

It is evident that lower total energy values translate to lower cohesive energy values. For example, the total energy for the MoS_2_ substrate is −575.12 eV, resulting in cohesive energy values of −49.81 eV for the c-BN configuration and −56.08 eV for the h-BN configuration. Moreover, the cohesive energy values of c-BN and h-BN on the Ni substrate are −45.66 eV and −57.16 eV, respectively, while the total energy of the Ni substrate is −404.09 eV.

#### 2.1.3. Cohesive Energy

**Figure 3:**
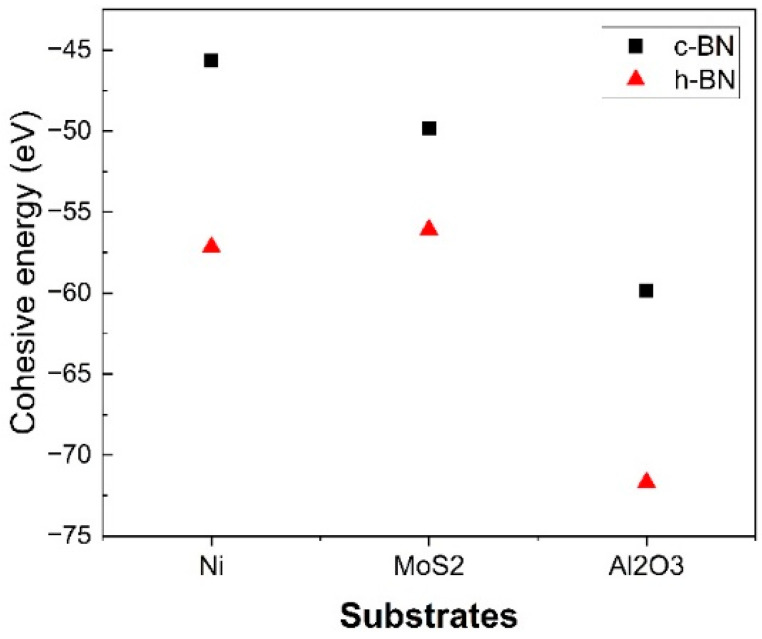
Cohesive energy values of c-BN and h-BN growth on different substrates revealed by using DFT calculations.

The calculation of cohesive energy, $E_{coh}$, is one of the main targets of this work. Figure 3 illustrates cubic boron nitride (c-BN) and hexagonal boron nitride (h-BN) growth behavior on various substrates, including Ni, MoS_2_, and Al_2_O_3_. The graph’s black squares represent the cubic boron nitride structure, while the red triangles correspond to the hexagonal boron nitride structure. Computational results reveal that the cohesive energy of h-BN when grown on a Ni substrate is −57.16 eV, significantly lower than the cohesive energy of c-BN at −45.66 eV. This trend persists for h-BN growth on the MoS_2_ and Al_2_O_3_ substrates, where the cohesive energy values remain lower than c-BN grown on the same substrates. Consequently, these findings underscore the greater stability of the h-BN structure in comparison with c-BN, suggesting that h-BN is more likely to grow on substrates under stable conditions.

### 2.2. Validation of DFT Calculations

Once DFT calculations have been executed, the subsequent validation phase becomes crucial. This phase extends beyond merely calculating energy levels and atomic arrangements, also encompassing the evaluation of experimentally measurable properties, like cohesive energy. While deviations between computational predictions and experimental data might be due to shortcomings in the DFT methodology, they more commonly reflect variances between the conditions under which computational and experimental analyses are conducted. For example, unaccounted impurities in the experimental setup could influence the results, or kinetic limitations may affect the observed outcomes. Additionally, the experimental findings could diverge from computational predictions due to other poorly represented factors in translating the problem from a theoretical to an empirical context. Nonetheless, it is vital to underline that one of the key benefits of computational modeling, particularly in DFT, is its ability to furnish data that are either elusive or notably challenging to capture through traditional lab-based experiments. Subsequently, the investigation of BN nanodots on Ni and MoS_2_ substrates was conducted via FE-SEM (Field-Emission Scanning Electron Microscopy), AFM (Atomic Force Microscopy), Raman spectroscopy, and TEM (Transmission Electron Microscopy) after growth resulting from using the PA-MBE technique.

#### 2.2.1. FE-SEM Analysis of BN on Ni

**Figure 4:**
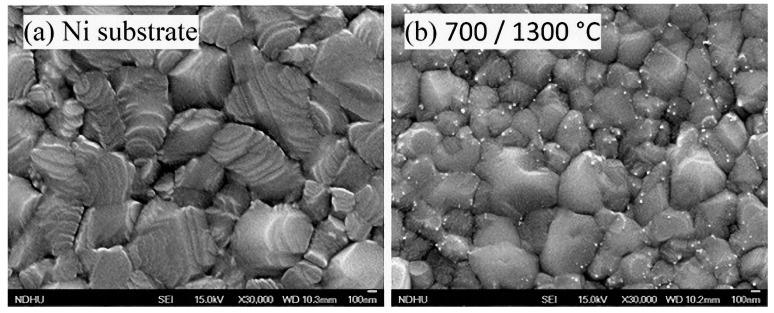
FE-SEM images: (**a**) polycrystal Ni substrate; (**b**) BN nanodots grown on Ni substrate.

The surface morphology of the specimens underwent meticulous examination through the utilization of FE-SEM. As depicted in Figure 4a, this examination unveiled a polycrystalline nickel film characterized by a rugged surface marked by a profusion of grain boundaries. In contrast, Figure 4b provides insight into the growth results conducted at a substrate temperature of 700 °C, with a K-cell temperature of 1300 °C. This image distinctly portrays a polycrystalline nickel substrate adorned with discernible white spots, representing the presence of BN nanostructures. The analysis of the collected data showed that the BN nanodots displayed an average diameter of approximately 40 nm. The proclivity for the formation of BN nanostructures along the grain boundaries of the nickel films can be attributed to the underlying mechanism of edge growth and the minimization of surface energy [41,42]. It is plausible to infer that the nucleation of h-BN initiated heterogeneously, subsequently leading to the formation of islands during the nascent stages of growth. The grain boundary of Ni played the role of a preferential nucleation site for the growth of BN. This phenomenon also finds substantiation in concurrent Raman spectral data.

#### 2.2.2. Raman Spectra Analysis of Ni and BN on Ni

**Figure 5:**
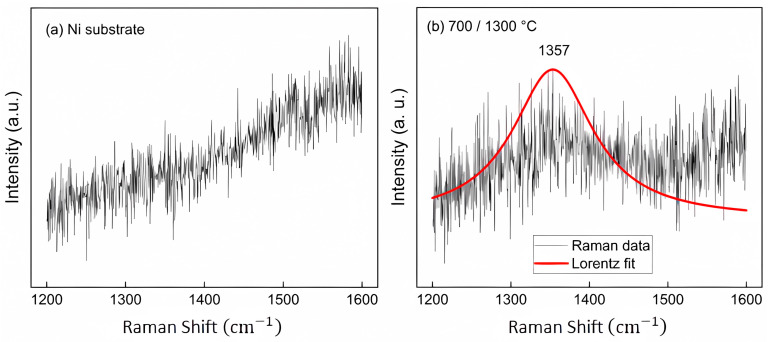
Raman spectra: (**a**) Ni substrate and (**b**) h-BN grown on Ni.

The elucidation of BN NDs’ vibration modes was achieved by using Raman spectroscopy. Figure 5a presents the Raman spectrum of the substrate. Subsequently, Figure 5b highlights the presence of a broad peak, signaling the existence of h-BN. The Raman spectra underwent Lorentzian fitting to gain a more comprehensive understanding, revealing a distinct Raman shift at 1357 cm^−1^ for h-BN within the sample. This observation is compelling evidence of crystalline h-BN’s presence on the nickel substrate. It is essential to recognize that the vibrational bonding mode is intricately governed by crystal symmetry, and the selection rules dictate optical activity under various excitation sources [43]. In the context of c-BN, the single, triply degenerate phonon exhibits IR and Raman activity. This phonon is further divided into a transverse optical (TO) component, located at approximately 1065 cm^−1^, and a longitudinal optical (LO) component, situated at around 1310 cm^−1^, owing to the ionic nature of the crystal. In contrast, for h-BN, two infrared active modes manifest with TO frequencies at approximately 1380 cm^−1^ and 780 cm^−1^ [44].

#### 2.2.3. AFM Analysis of BN on MoS_2_

**Figure 6:**
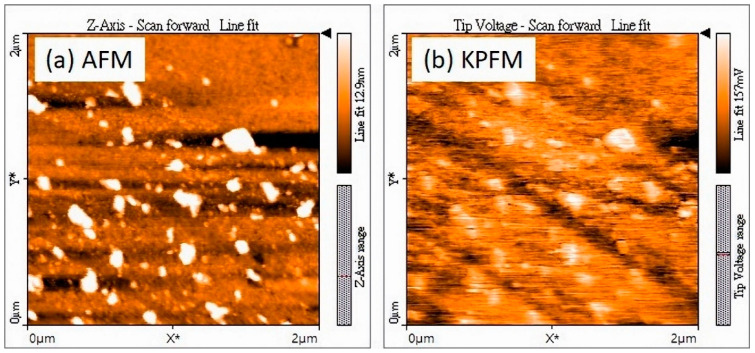
(**a**) AFM and (**b**) KPFM images of BN grown on MoS_2_.

In the second case, corresponding to sample S2 in Table 3 of Section 3.2, BN was grown on a few-layer 2D MoS_2_ prepared by the chemical vapor deposition technique. Because of the semiconducting nature of the MoS_2_ layer, AFM was employed to observe the surface morphology after the growth. Nanodot structures were clearly observed in an AFM image with a scanning area of 2 µm × 2 µm, shown in Figure 6a. Meanwhile, an image taken using Kevin Probe Force Microscopy (KPFM), performing contact potential distribution as a work function difference, confirmed that the BN nanodot structures grew on the surface of the MoS_2_ substrate as shown in Figure 6b.

#### 2.2.4. Raman Analysis of BN on MoS_2_

**Figure 7:**
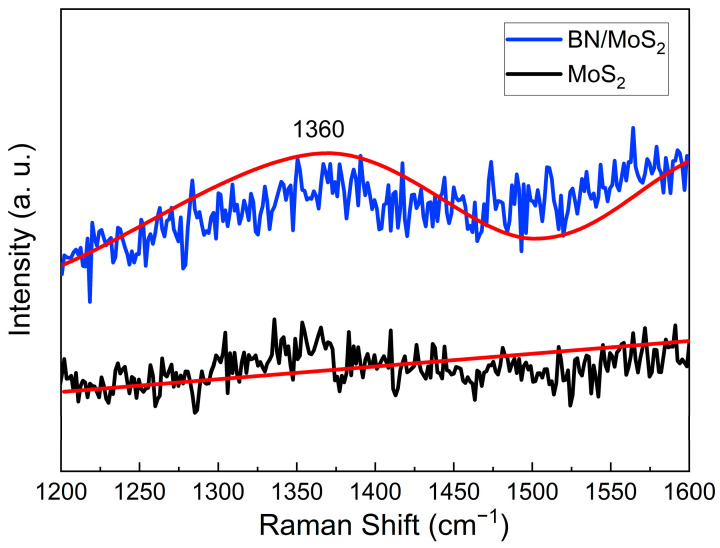
Raman spectra for BN/MoS_2_ and MoS_2_ substrates. The blue line represents the Raman data for BN growth on the MoS_2_ substrate, while the black line corresponds to Raman data for the MoS_2_ substrate. The red lines indicate the Lorentz fits for both spectra.

For the analysis of sample S1, Raman spectra were utilized to characterize the vibration mode of the BN QDs and substrate. Figure 7 shows the Raman spectra of MoS_2_ and BN on MoS_2_ within a range of 1200 and 1600 cm^−1^. After the Lorentzian fitting of the spectra, it reveals a distinct Raman shift at 1360 cm^−1^ for the h-BN phase on the substrate of MoS_2_. The typical MoS_2_ spectra were observed at wavenumbers of ~387.4 cm^−1^ and ~409.2 cm^−1^, corresponding to the atomic vibration modes of E_2g_ and A_1g_, respectively [45]. Therefore, we cannot observe any peak for MoS_2_ in the spectra within a range of 1200 and 1600 cm^−1^.

#### 2.2.5. TEM Analysis of BN on MoS_2_

**Figure 8:**
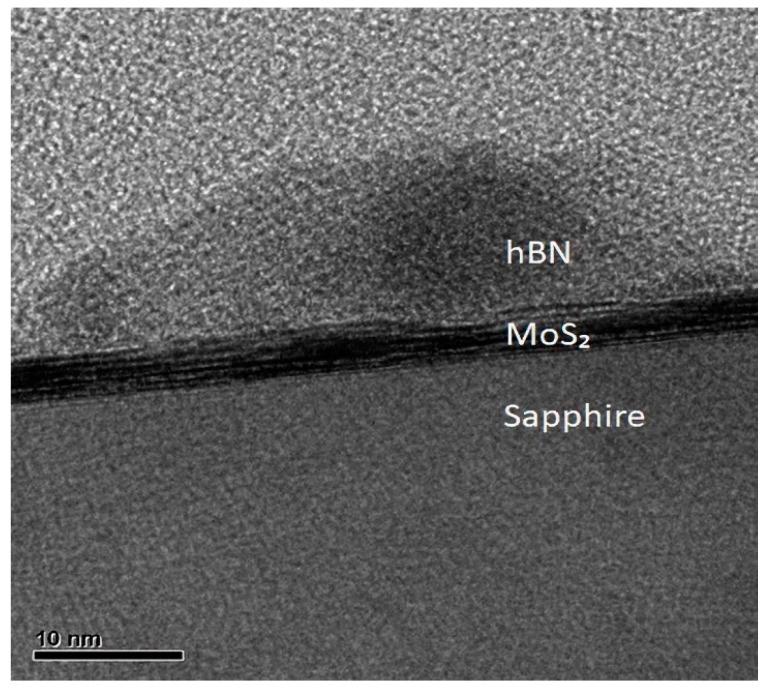
TEM image for BN grown on MoS_2_.

To further investigate the h-BN nanodots on the surface of the few-layer 2D MoS_2_, TEM was used to observe the nanodots of BN after the sample was prepared by using the focus ion beam technique. Figure 8 shows a cross-sectional TEM image of sample S2, including one nanodot of h-BN, the few-layer MoS_2,_ and the sapphire substrate. The average size of the h-BN nanodots was estimated to be approximately 40 nm, too. BN nanodots were grown from the single-crystal MoS_2_ layer via the mechanism of absorption, the diffusion of ad-atoms, cluster nucleation, and the aggregation of nanodots [46]. In the epitaxial growth of the lattice-mismatched heterostructure system of h-BN (lattice constant, a = 2.504 Å) on MoS_2_ (lattice constant, a = 3.15 Å), the epitaxial growth mode could belong to the Volmer–Weber kinetic model [47].

In the two experiments, h-BN exhibited more stability and more easily grew on the Ni and MoS_2_ substrates compared with c-BN. The nanodots of h-BN were realized using the PA-MBE technique, underscoring the promising future of DFT calculations in materials science. Future advancements in this field will involve the development of more accurate and versatile exchange–correlation functionals that can better handle complex systems, including strongly correlated materials and those with intricate electronic structures. The integration of machine learning and data-driven approaches will enhance the speed and precision of material property predictions, accelerating materials discovery processes. Furthermore, the advent of quantum computing holds promise for providing a quantum advantage, allowing for unprecedented complexity and accuracy simulations. In addition to fundamental research, DFT will play a pivotal role in designing and optimizing materials for sustainable energy solutions, environmental remediation, and advanced technologies. Collaborations between computational scientists, experimentalists, and researchers from various domains will drive interdisciplinary breakthroughs, further expanding the capabilities and impact of DFT in materials research.

## 3. Materials and Methods

### 3.1. DFT Calculation Method

DFT is a versatile computational tool that offers various approaches for approximating solutions to the many-body Schrödinger problem. It employs density-functional theory to tackle Kohn–Sham (KS) equations and Hartree–Fock (HF) approximation for Roothaan equations [48]. DFT encompasses a range of techniques, including the GW method, random-phase approximation, and Schrödinger equations [49].

(1) ∈Ψ=H^Ψ

Ψ represents the wave function, ∈ denotes energy, and H signifies the Hamiltonian operator, which is an integral component of the many-body Schrödinger equation, represented by Equation (1), where the Hamiltonian operator acts on a specific wave function to yield a proportional energy result, elucidating a stationary state. The Schrödinger wave equation for a single particle moving in an electric field is expressed as [49]

(2) $$E\Psi \left(r\right)=\left[\left(\frac{{-h}^{2}}{2m}\right)\nabla^{2}+V\left(r\right)\right]\Psi (r)$$

and the time-independent Schrödinger equation is [47]

(3) $$H\Psi =\left[T+V+U\right]\Psi =E\Psi$$

This further elucidates the interplay between the kinetic energy (*T*), potential energy (*V*), and interaction energy (*U*), culminating in the total energy (*E*). In the realm of computational materials science, particularly in the Vienna ab initio simulation package (VASP), plane-wave basis sets play a pivotal role, facilitating the representation of crucial parameters such as single-electron orbitals, electronic charge density, and the local potential. The interactions between electrons and ions are aptly described through norm-conserving or ultra-soft pseudopotentials or, alternatively, the projector-augmented wave method.

In this study, the energies obtained from the DFT calculations were employed to predict cohesion energy. The methodology entailed the initial relaxation of a substrate’s unit cell, followed by the construction of a 3 × 3 × 3 supercell utilizing the relaxed unit cell. Similarly, unit cells of boron nitride (BN) in hexagonal and face-centered cubic structures underwent relaxation.

Subsequently, supercells of BN were created, and energies of both supercells and substrates were calculated. A combination of hexagonal and face-centered cubic BN with various substrates was prepared, allowing for the relaxation of the final structure by permitting the movement of two layers of substrate and BN atoms. Cohesive energy (*E_coh_*), crucial in assessing crystal stability, was then computed according to Equation (4):

(4) $$E_{coh}=(E_{deposited})-E_{substrate}-E_{BN\;nanodots}$$

where $E_{deposited}$ denotes the total energy of different substrates with boron nitride (BN). $E_{substrate}\;$denotes the total energy of the substrate (i.e., Ni, MoS_2_, or Al_2_O_3_) before deposition. $E_{BN\;nanodots}$ signifies the total energy of the cubic and hexagonal BN structures before deposition. $E_{coh}$ represents the energy by arranging the BN crystal on different substrates, as compared with pure BN and substrates, which is related to the force binding the crystal and substrate together [50].

The outcome of the aforementioned calculations inherently relies on the sizes of the supercells utilized, necessitating the interpretation of results as qualitative indications of structural stability. Throughout all the calculations, a cutoff energy was set to 520 eV. A convergence test with respect to k-points was performed before simulation, and the k-point density was set to 0.1 eV/Å, which allowed us to keep the result within an accuracy of 1 × 10^−3^ eV.

In real-world experiment settings, the deposition of materials onto substrates commonly occurs within a vacuum environment to mitigate interactions with extraneous particles, thereby bolstering result fidelity. Similarly, within the context of DFT calculations, the deposition of materials such as boron nitride (BN) onto different substrates can also be modeled under vacuum conditions; the size was set at 13 Å. Prior to these calculations, it is essential to conduct atomic optimization, which entails fine-tuning the atomic positions and electronic structure to identify the system’s most stable configurations [51]. This optimization process includes identifying the arrangement of atoms or particles that correspond to the minimum potential energy.

### 3.2. Experimental Method

**Figure 9:**
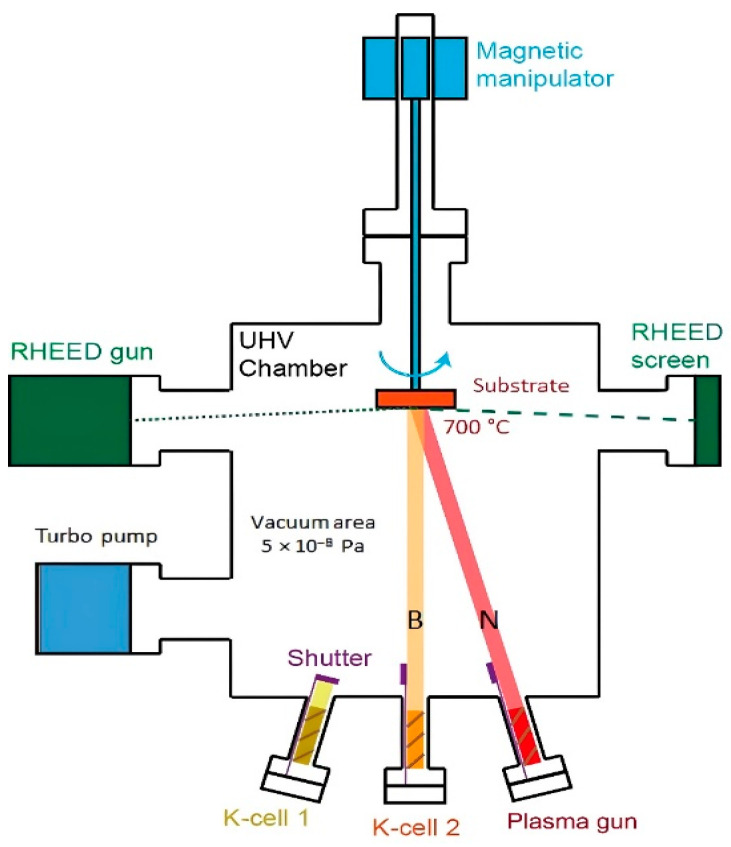
Schematic of the processing BN NDs grown on Ni and MoS_2_ substrates with the PA-MBE system.

To prove the prediction of the stability of BN growth on different substrates by using the DFT described above, we proceeded with experimental validation for two kinds of substrates, Ni and MoS_2_. The schematic of the experimental process for PA-MBE is shown in Figure 9. The system parameters we employed in this experiment are as follows: The synthesis of boron nitride nanodots was achieved by using a ULVAC PA-MBE system. A boron source in the form of a high-purity slug (99.9999% purity) was introduced into the growth chamber in a high-temperature K-cell. Nitrogen gas of 99.9999% purity, flowing at a rate of 0.8 sccm, served as the nitrogen plasma source, while radio frequency power was maintained at 500 W. The substrate was maintained at a growth temperature of 700 °C, preceded by a thermal cleaning step to eliminate surface moisture and oxides. The base working pressure (vacuum) was maintained at 5 × 10^−8^ Pa, and the K-cell temperatures were set to 1300 or 1460 °C, facilitating the concurrent generation of boron during the growth process. Growth parameters for the two samples are shown in Table 3. After the growth, the samples were gradually cooled to an ambient temperature within the controlled environment of the growth chamber.

### 3.3. Characterization of BN Nanodots

The growth process of BN nanodots was monitored by using 20 kV in situ reflection high-energy electron diffraction (RHEED). After the growth, the surface morphology was analyzed by utilizing a JEOL JSM-7000F FE-SEM (Akishima, Japan) equipped with silicon–drift–detector-based energy dispersive X-ray spectroscopy (EDS). Surface roughness and local contact voltage, indicating the material work functions, were quantified by using AFM and KPFM (Nanosurf C3000, Liestal, Switzerland), respectively. Additionally, Raman spectroscopy (Renishaw, Shinjuku, Japan), employing a 532 nm laser with a power of 2 mW, was employed to analyze the h-BN QDs for 10 s with a spot size of around 1 µm. Transmission electron microscopy (JEOL JEM-2100F CS) was utilized to observe the nanodots of BN, and a TEM sample was prepared with a focus ion beam system (FEI Helios G3CX, Thermo Fisher Scientific, Waltham, MA, USA).

**Figure 10:**
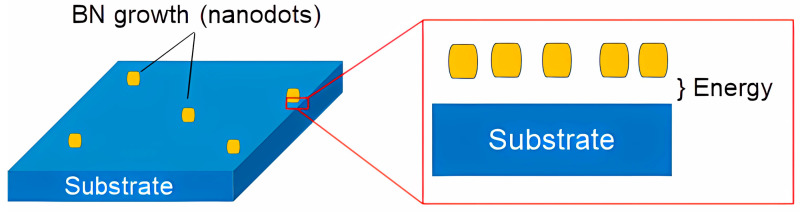
Illustrates the BN nanodots’ growth on a substrate and cohesive energy in between.

Further, for the calculation approach, the growth conditions for both c-BN and h-BN NDs on different Ni, MoS_2_, and Al_2_O_3_ substrates were simulated as experimental conditions, as illustrated in Figure 10.

## 4. Conclusions

We successfully designed and executed DFT calculations for the deposition of BN with both FCC and HCP structures on Ni, MoS_2_, and Al_2_O_3_ substrates. In addition, two experimental validations have been proposed, specifically confirming the deposition of h-BN nanodots on Ni and MoS_2_ substrates. A comprehensive synthesis of our findings and their implications follows.

1. The DFT calculations demonstrate a robust agreement with the experimental data, affirming the superior stability of h-BN as the most favorable crystal structure.
2. DFT proves its capability in predicting the stability of boron nitride growth across various substrates, providing valuable insights into potential applications.
3. The epitaxial growth of h-BN is established as a viable process on the Ni and 2D MoS_2_ surfaces, showing PA-MBE to be a promising technique.
4. Detailed characterization through FE-SEM, Raman spectroscopy, AFM, and TEM confirms the successful formation of h-BN nanodots on Ni and MoS_2_ surfaces.
5. Our findings underscore the significant potential of MBE growth for h-BN NDs, offering an adaptable approach with promising prospects for the optoelectronic applications of h-BN.

## Figures and Tables

**Table 1:** Atom composition of each element in different configurations of c-BN and h-BN growth on different substrates (Ni, MoS_2_, and Al_2_O_3_).

Configuration/Atom	B	N	Ni	Mo	S	Al	O
c-BN/Ni(111)	8	8	78				
h-BN/Ni(111)	8	8	78				
c-BN/MoS_2_(111)	8	8		27	52		
h-BN/MoS_2_(111)	8	8		27	52		
c-BN/Al_2_O_3_(111)	8	8				40	57
h-BN/Al_2_O_3_(111)	8	8				40	57

**Table 2:** The total energy and energy cohesive BN growth on the different substrates.

	Total Energy (eV)			Cohesive Energy (eV)	
Structure	Pure	c-BN Growth	h-BN Growth	c-BN Growth	h-BN Growth
c-BN	−44.54	-	-	-	-
h-BN	−49.10	-	-	-	-
Ni	−404.09	−494.29	−510.35	−45.66	−57.16
MoS_2_	−575.12	−669.47	−680.30	−49.81	−56.08
Al₂O₃	−622.00	−726.36	−742.79	−59.82	−71.69

**Table 3:** Summary of the growth parameters of BN nanodots in the PA-MBE system.

Sample	Substrate	Thermal Cleaning	Growth Temperature	Pre-Boron Treatment	Temperature of Boron Cell	Growth Duration
S1	Ni	600 °C (30 min)	700 °C	20 min	1300 °C	120 min
S2	MoS_2_	300 °C (10 min)	700 °C	20 min	1460 °C	60 min

## Data Availability

The data presented in this study are available at the request of the corresponding authors.
